# Downregulation of circ-UBAP2 ameliorates oxidative stress and dysfunctions of human retinal microvascular endothelial cells (hRMECs) via miR-589-5p/EGR1 axis

**DOI:** 10.1080/21655979.2021.1979440

**Published:** 2021-10-05

**Authors:** Yu Jiewei, Zhou jingjing, Xue jingjing, Zhang Guilan

**Affiliations:** a Ophthalmology Department, Jiujiang Hospital of Traditional Chinese Medicine, Jiujiang City, Jiangxi Province, China; b Ophthalmology Department, Affiliated Hospital of Jiangxi University of Traditional Chinese, Nanchang City, Jiangxi Province, China; c Ophthalmology Department, The Third Clinical Medical College of China Three Gorges University, Gezhouba central hospital of sinopharm, Yichang City, Hubei Province, China

**Keywords:** Diabetic retinopathy, circ-UBAP2, miR-589-5p, early growth response 1, angiogenesis, oxidative stress

## Abstract

Hsa_circ_0001850_circ_0001850 (circ-UBAP2) is reported to be upregulated in diabetic retinopathy (DR). However, its role in high glucose (HG)-triggered oxidative stress and vascular dysfunction in DR is unclear. This study aimed to investigate the potential of circUBAP2 in DR. The content of malondialdehyde (MDA), and the activities of superoxide dismutase (SOD) and glutathione peroxidase (GSH-PX) were analyzed using the corresponding kits. Western blotting was performed to detect the protein expression of Nrf2, HO-1, and SOD-1. MTT assay was conducted to assess cell viability. A transwell migration assay was used to determine the migration ability of human retinal microvascular endothelial cells (hRMECs). A Matrigel tube formation assay was performed to analyze tube formation. The targeting relationships were verified using a luciferase reporter assay. We found that the circ-UBAP2 expression increased in DR patients and HG-treated hRMECs. Downregulation of circ-UBAP2 ameliorated HG-induced oxidative stress and dysfunction of hRMECs. Mechanistically, circ-UBAP2 sponges miR-589-5p, which is downregulated under hyperglycemic conditions. In addition, EGR1 was confirmed to be a target gene of miR-589-5p and was overexpressed in HG-treated hRMECs. In addition, EGR1 reversed the effects of miR-589-5p and induced oxidative stress and dysfunction in hRMECs. Taken together, knockdown of circ-UBAP2 relieved HG-induced oxidative stress and dysfunctions of the hRMECs through the miR-589-5p/EGR1 axis, which may offer a promising therapeutic target for DR.

## Introduction

Diabetic retinopathy (DR), a microvascular complication of diabetes mellitus, is the primary cause of blindness in patients with diabetes [1]. The initiation and development of DR are closely associated with chronic retinal exposure to hyperglycemia [2]. Hence, the main research focus for treating proliferative diabetic retinopathy (PDR) will be to explore the molecular mechanisms underlying the occurrence and development of PDR for ameliorating oxidative stress, abnormal cell proliferation, and retinal neovascularization [3]. The pathogenesis of DR is complicated, including high expression of growth factors, accumulation of glycosylation end products, and oxidative stress [4]. Under hyperglycemia, intracellular glucose metabolism and reactive oxygen species (ROS) content are increased, while superoxide dismutase (SOD) and glutathione peroxidase (GSH-PX) activities are reduced [5]. A large number of ROS accumulate abnormally in the retina generate chronic damage to the retinal tissue, leading to the occurrence of DR [6]. Therefore, exploring the potential molecular mechanisms of the occurrence and development of PDR to ameliorate oxidative stress, abnormal cell proliferation and neovascularization of the retina are the main research directions for the treatment of PDR.

Circular RNAs (circRNAs), a novel type of non-coding RNAs with closed-loop structure, are abundantly and stably expressed in eukaryotes and participate in various cell biological behaviors [7]. Presently, with the increasing investigations on the biological roles of circRNAs, the functions can be summarized as follows [1]: complementary binding with miRNAs to regulate mRNAs [2], regulation of the expression and transcription of host genes, and [3] competition with linear RNAs to regulate the classic splicing process of RNAs [8]. Emerging evidence has emphasized the promising effects of circRNAs in the diagnosis and prognosis of DR [9]. Depletion of circCOL1A2 suppresses proliferation, migration, angiogenesis and vascular permeability of hRMECs via promoting the level of miR-29b [10]. Overexpression of circDNMT3B reduced the number of retinal acellular capillary as well as attenuated visual impairment in diabetic rats [11]. He et al demonstrate that hsa_circ_0001850 (circUBAP2) is significantly upregulated in DR [12]. However, the underlying mechanisms of action of hsa_circ_0001850 in DR have not been elucidated.

Here, we hypothesis that circUBAP2 may play a critical role in the progression of DR. The aim and goal of this study was to explore the biological function and molecular mechanism of circUBAP2 in oxidative stress and angiogenesis induced by high glucose in human retinal microvascular endothelial cells (hRMECs).

## Materials and methods

### Sample collecting

Vitreous humor samples were collected from DR patients (n = 21) and healthy people (n = 21) from 23 March 2018, to 3 July 2019, at the First Affiliated Hospital of Fujian Medical University. All enrolled patients were diagnosed with DR at the ophthalmology department of the hospital. Patients who were underaged (<18 years), transferred from other hospitals, suffering from cancer or hematologic malignancies, or pregnant or lactating were excluded from this study. Informed consent was obtained from all the enrolled donors. Ethical approval was obtained from the Ethics Committee of the First Affiliated Hospital of Fujian Medical University. All experiments strictly followed the principles of the Declaration of Helsinki.

### Cell culture and transfection

Human retinal microvascular endothelial cells (hRMECs) were obtained from Tongpai Biotech Co., Ltd and cultured in the Dulbecco’s Modified Eagle Medium (DMEM) with 10% FBS, 100 U/ml streptomycin and 100 U/ml penicillin (all obtained from Beyotime Biotech Co., Ltd.) at 37°C in the presence of 5% CO_2_. After the cell confluence reached 70%-80%, the cells were collected for the subsequent experiments.

Cells were divided into normal glucose (NG) and high glucose (HG) groups. The glucose concentrations of DMEM were adjusted to 5.5 mM (NG) and 25 mM (HG).

Cells in the logic growth phase were transfected with small interfering RNA of circ-UBAP2 (si-circ-UBAP2: 5ʹCTGGACTGAAGATGATTTGGA3ʹ), miR-589-5p inhibitor, miR-589-5p mimic, pcDNA3.1/EGR1, and the corresponding negative controls (all designed and synthesized by Fenghui Biotech Co., Ltd.) with the concentration of 100 nM using Lipofectamine 3000 (Thermo Fisher) according to the manufacturer’s instructions. After 48 h, the cells were used for subsequent experiments [10].

Fluorescence in situ hybridization (FISH) assay

Alexa Fluor 488 labeled circ-UBAP2 and Alexa Fluor 555-labeled miR-489-5p probes were designed and synthesized by RiboBio (Guangzhou, China). FISH experiment was carried out with a Fluorescence in situ Hybridization Kit (RiboBio, Guangzhou, China). 1 × 105 cells were seed onto autoclaved glass slides and cultured for 24 h. After fixation with 4% paraformaldehyde for 20 min, permeabilization was done with 0.5% Triton X-100 for 10 min, and the cells cultured at 37°C overnight. Finally, the slides were incubated with DAPI and observed under a fluorescence microscope (Leica, Wetzlar, Germany).

### Real-time quantitative polymerase chain reaction (RT-qPCR) assay

TRIzol (Invitrogen) was added into the hRMECs and the vitreous humor samples to extract the total RNA. cDNAs were obtained from the RNA reverse transcription. Afterward, RT-qPCR was conducted followed SuperSYBR One Step RT-qPCR Kit (High ROX) manual (WE0139; Biolab Co., Ltd.) using the ProFlex PCR System (Thermo Fisher). The expression of mRNA and miRNA were analyzed with the 2-ΔΔCt method. GAPDH and U6 were used as internal references for mRNA and miRNA, respectively [10]. The primer sequences were as follows: circ-UBAP2, forward5ʹ-TGTGGAAGAGTGGACAACAGA-3ʹ, and reverse 5ʹ-TTGAGAACCTTCCTGCCCC-3ʹ; miR-589-5p forward 5ʹ-CGAGGTCAGCGTGATTTCA TGG-3ʹ and reverse 5ʹ-TGTGTCCAAGTCCCAGCCAGAG-3ʹ; EGR1 forward 5ʹ-AGCCCTACGAGCACCTGAC-3ʹ and reverse 5ʹ-GGTTTGGCTGGGGTAACTG-3ʹ; GAPDH forward 5ʹ-TGAAGGTCGGAGTCAACGGATTTGGT-3ʹ and reverse 5ʹ-CATGTGGGCCATGAGGTCCACCAC-3ʹ; U6 forward 5ʹ-GCTTCGGCAGCACATATACTAAAAT-3ʹ and reverse 5ʹ-CGCTTCACGAATTTGCGTGTCAT-3ʹ.

### MTT assay

Cells were resuspended in PBS at the density of 1 × 10^4^ cells/ml and added into the 96-well plate at density of 100 μl/well. Then cells were incubated with 100 μl MTT solution (BTN111105; Biolab Co., Ltd.) under the conditions of 37°C, 5% CO_2_ for 4 h. Afterward, 200 μl dimethyl sulfoxide solution (Macklin Biochemical Co., Ltd.) was used to resolve formazan crystals. The optical densities were measured using a microplate reader at the wavelength of 490 nm [11].

### Determination of the oxidative stress biomarkers

The cells were lysed and centrifuged for detecting the amounts of MDA (S0131S; Beyotime), SOD (S0101S; Beyotime), and GSH-PX (ML5065; Shanghai MlBio) using the corresponding kits according to the manufacturer’s protocols [11].

### Transwell migration assay

The cells were collected and rinsed twice with pre-chilled PBS. Then, the cell density was adjusted to 1 × 10^5^ cells/ml in serum-free DMEM. Transwell chambers (3421; Corning) were inserted into a 24-well plate. Basolateral chambers contained a medium with 10% FBS, and the apical chambers contained the cell suspension. After two days, the migrated cells were fixed with 100% methanol for 30 min, stained with 0.1% crystal violet (both from Solarbio Technology Co., Ltd.), and observed under an inverted microscope (CKX53; Olympus Optical Co., Ltd.) at a magnification of 200 × . Five random visual fields of each well were captured using a light microscope [10].

### Tube formation assay

Tube formation of hRMECs was analyzed as previously described [13]. Briefly, hRMECs were seeded into a 24-well plate pre-coated with Matrigel (356,234; Corning) (300 μl). After 24 h of incubation, the tube-like structures were photographed under an inverted microscope. Five random visual fields were observed for each well. ImageJ software (version 1.8.0) was used to count the tube-like structures.

### Western blot analysis

The total proteins of hRMECs were extracted using RIPA lysis buffer (C1053-100; Applygen Biotech Co., Ltd.) for 30 min. Protein concentration was quantified using the BCA Protein Assay Kit (KTD3001; Abbkine). Proteins were separated by 10% sodium dodecyl sulfate polyacrylamide gel electrophoresis (SDS-PAGE) for 1.5 h at 120 V. Then, the separated proteins were transferred onto Polyvinylidene fluoride (PVDF) membranes (Millipore) for 2 h at 200 mA. After blocking with blocking buffer for 1 h, the membranes were incubated overnight with primary antibodies, such as anti-Nrf2 (1:500; A-ALS14857; Fitzgerald), anti-HO-1 (1:1000; A-AJ1338b; Abgent), anti-SOD-1 (1:200; 6641–30 T; Biovision), and anti-GAPDH (1:3000; 10 R-10262; Fitzgerald) at 4°C. The next day, the membranes were incubated with a goat anti-human IgG secondary antibody (1:4000; 70–1097; Fitzgerald) at 25°C for 2 h. Proteins were analyzed using an Ultra High Sensitivity ECL Kit (GK10008; Glpbio). GAPDH was used to normalize protein expression [11].

### Dual-luciferase reporter assay

The dual-luciferase reporter vectors of wild-type (WT) and mutant (MUT) 3´-UTR circ-UBAP2 or EGR1 were co-transfected into the hRMECs with miR-589-5p mimic/NC mimic (all synthesized by GenePharma Co., Ltd.) and incubated for 24 h. The results were determined using a luciferase reporter kit (ZY130595; Zeye Biotech Co., Ltd.). Luciferase activity was normalized to *Renilla* luciferase activity [13].

## Statistical analyses

Eac independent experiment was conducted for three times. All data were analyzed using GraphPad Prism (version 8.4.3.686; GraphPad Software, Inc.) and presented as mean ± SD. The Student’s t-test was used to measure the statistical differences between the two groups. ANOVA was applied to multiple groups. Statistical significance was set at *P* < 0.05.

## Results

We hypothesis that circUBAP2 may play a critical role in the progression of DR. Here, a various of experiments were performed to to explore the biological function and molecular mechanism of circUBAP2 in oxidative stress and angiogenesis induced by high glucose in human retinal microvascular endothelial cells (hRMECs).

### Circ-UBAP2 overexpression in DR patients and HG-treated hRMECs

**Figure 1.**
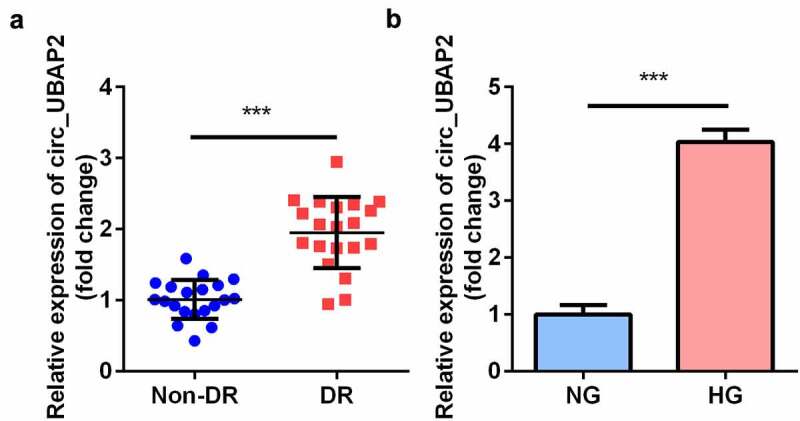
**The expression levels of circ-UBAP1 in human vitreous humor and hRMECs**. (a) Vitreous humor samples were collected from DR patients (n = 30) and non-DR people (n = 30) to analyze the expression of circ-UBAP1 using RT-qPCR. (b) circ-UBAP2 expression was measured after hRMECs were treated with HG or NG. Each experiment was performed in triplicate. ***P < 0.001. DR, diabetic retinopathy; hRMECs, human retinal microvascular endothelial cells; HG, high glucose; NG, normal glucose

RT-qPCR results demonstrated that the expression of circ-UBAP2 in the vitreous humor samples from DR patients was notably higher than that in healthy individuals (Figure 1a). Furthermore, in the DR cell model, HG treatment significantly elevated the level of circ-UBAP2 in hRMECs (Figure 1b).

### Downregulation of circ-UBAP2 relieved HG-induced OS in the hRMECs

**Figure 2.**
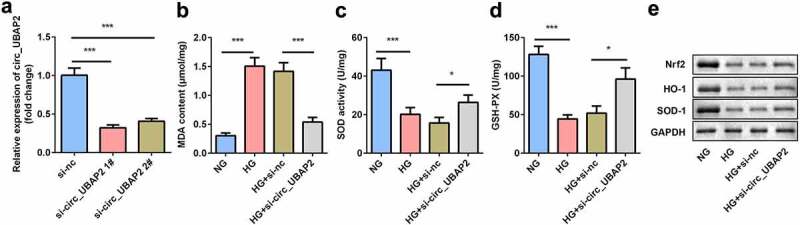
**Knockdown of circ-UBAP2 attenuated oxidative stress triggered by HG in hRMECs**. (a) The transfection efficiency of circ-UBAP2. (b) MDA contents, (c) SOD, and (d) GSH-PX activities in hRMECs were detected using the corresponding kits after the indicated treatments. (e) Western blotting for the proteins of Nrf2, HO-1, and SOD-1. GAPDH was used to normalize the protein levels. Each experiment was performed in triplicate. *P < 0.05; ***P < 0.001. MDA, malondialdehyde; SOD, superoxide dismutase; GSH-PX, glutathione peroxidase; hRMECs, human retinal microvascular endothelial cells

The expression of circ-UBAP2 was markedly decreased by si-circ-UBAP2 (Figure 2a). The transfection efficiency was more potent in the si-circ-UBAP2 1# group, which was then used in subsequent experiments. The malondialdehyde (MDA) concentration was significantly increased by HG treatment, which was antagonized by si-circ-UBAP2, leading to a decreased MDA (Figure 2b). Inversely, HG reduced SOD and GSH-PX activities, while si-circ-UBAP2 reversed the effects of HG treatment (Figure 2c and d). Moreover, the protein expression of anti-OS genes was examined. The downregulation of Nrf2, HO-1, and SOD-1 induced by HG was abrogated by si-circ-UBAP2 (Figure 2e).

### Knockdown of circ-UBAP2 inhibited viability, migration, and tube formation of the HG-treated hRMECs

**Figure 3.**
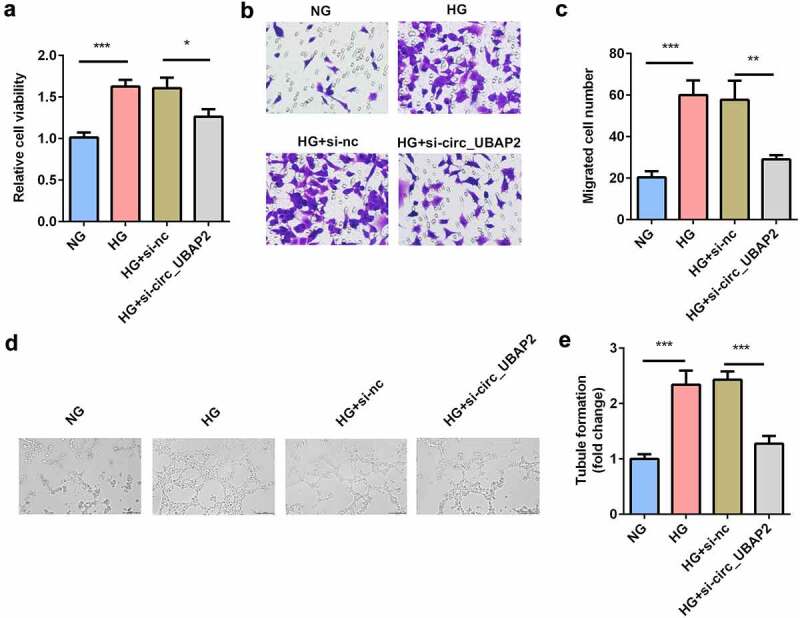
**Knockdown of circ-UBAP2 inhibited viability, migration, and tube formation of the HG-treated hRMECs**. (a) MTT assay was performed to determine the cell viability. (b) The migrated cells were fixed and stained after the transwell migration assay. (c) Quantification of B. (d) Images of the tube-like structures were observed under an inverted microscope after 24 h incubation. (e) Quantification of D. Each experiment was performed in triplicate. *P < 0.05; **P< 0.01; ***P < 0.001. HG, high glucose; MTT, 3-(4,5-dimethylthiazol-2-yl)-2,5-diphenyl tetrazolium bromide; hRMECs, human retinal microvascular endothelial cells

Next, the effects of si-circ-UBAP2 on cell viability, migration, and tube formation were evaluated. MTT results showed that cell viability of the HG-treated hRMECs was promoted, while knockdown of circ-UBAP2 inhibited cell viability (Figure 3a). Moreover, the number of migrated cells significantly increased under HG conditions (Figure 3b and c). However, si-circ-UBAP2 significantly reduced the number of migrated cells. HG treatment significantly accelerated tube formation, while si-circ-UBAP2 ameliorated tube formation in hRMECs (Figure 3e).

### Circ-UBAP2 sponged miR-589-5p

**Figure 4.**
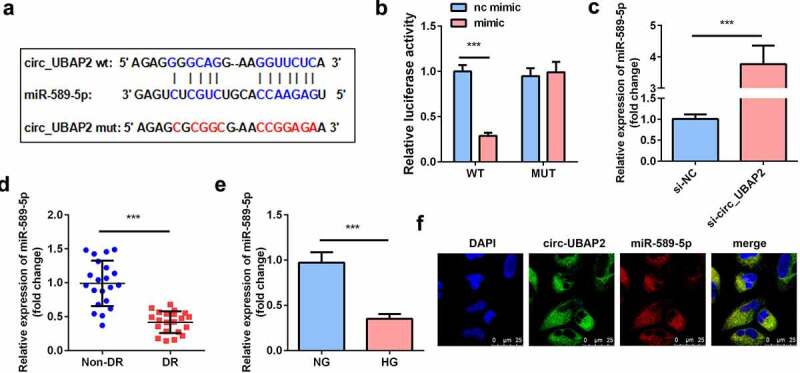
**miR-589-5p was sponged by circ-UBAP2**. (a) The binding sequences of miR-589-5p and circ-UBAP2. (b) Luciferase activities of hRMECs. (c) The expression level of miR-589-5p when the hRMECs were transfected with si-circ-UBAP2. (d) The expression levels of miR-589-5p in the vitreous humor samples from the DR patients (n = 21) and the non-DR people (n = 21). (e) The expression levels of miR-589-5p in hRMECs treated with NG or HG. (f) FISH was performed to detect the co-location between circ-UBAP2 and miR-589-5p. Each experiment was performed in triplicate. ***P < 0.001. WT, wild type; MUT, mutant type; DR, diabetic retinopathy; si-, small interference RNA; hRMECs, human retinal microvascular endothelial cells; HG, high glucose; NG, normal glucose

Bioinformatics analysis revealed the binding sequences of circ-UBAP2 and miR-589-5p (Figure 4a). In cells transfected with the circ-UBAP2 WT luciferase vector, miR-589-5p substantially decreased luciferase activity. However, no significant change was observed in cells transfected with circ-UBAP2 MUT and miR-589-5p/mimic NC (Figure 4b). Downregulation of circ-UBAP2 in hRMECs notably increased the expression of miR-589-5p, suggesting that circ-UBAP2 mechanically targets miR-589-5p (Figure 4c). In addition, the expression of miR-589-5p significantly decreased in DR patients and HG-treated hRMECs (Figure 4d, e). FISH assay was performed to examine the co-location of circ-UBAP2 and miR-589-5p. It was showed that there is co-location between circ-UBAP2 and miR-589-5p (figure 4f).

### Inhibition of miR-589-5p abrogated the effects of si-circ-UBAP2

**Figure 5.**
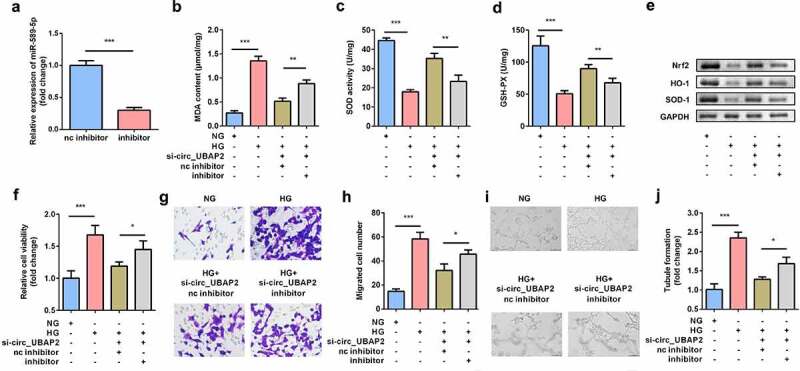
**Inhibition of miR-589-5p abrogated the effects of si-circ-UBAP2 on oxidative stress and dysfunctions of HG-treated hRMECs**. (a) The transfection efficiency of miR-589-5p. (b) MDA contents, (c) SOD, and (d) GSH-PX activities in the hRMECs were detected using the corresponding kits after the indicated treatments. (e) Western blotting was used to determine the protein expressions of Nrf2, HO-1, and SOD-1. GAPDH was used to normalize the protein expressions. (f) MTT assay was performed to determine the cell viability. (g) The migrated cells were fixed and determined using a transwell migration assay. (h) Quantification of G. (i) Images of tube-like structures were observed under an inverted microscope. (j) Quantification of I. Each experiment was performed in triplicate. *P < 0.05; **P < 0.01; ***P < 0.001. HG, high glucose; MTT, 3-(4,5-dimethylthiazol-2-yl)-2,5-diphenyl tetrazolium bromide; hRMECs, human retinal microvascular endothelial cells; MDA, malondialdehyde; SOD, superoxide dismutase; GSH-PX, glutathione peroxidase

To further investigate the relationship between miR-589-5p and circ-UBAP2, miR-589-5p was downregulated in circ-UBAP2-deficient hRMECs. The miR-589-5p inhibitor sharply reduced miR-589-5p levels in hRMECs (Figure 5a). MDA concentration was significantly decreased by circ-UBAP2 knockdown, while miR-589-5p inhibitor significantly increased MDA concentration (Figure 5b). Meanwhile, SOD and GSH-PX activities were reduced by the miR-589-5p inhibitor (Figure 5c and d). The protein levels of Nrf2, HO-1, and SOD-1 were also decreased by miR-589-5p (Figure 5e). Furthermore, inhibition of miR-589-5p abrogated the effects of si-circ-UBAP2 and promoted cell viability, migration, and tube formation in HG-treated hRMECs (figure 5f-j).

### EGR1 was the target gene of miR-589-5p

**Figure 6.**
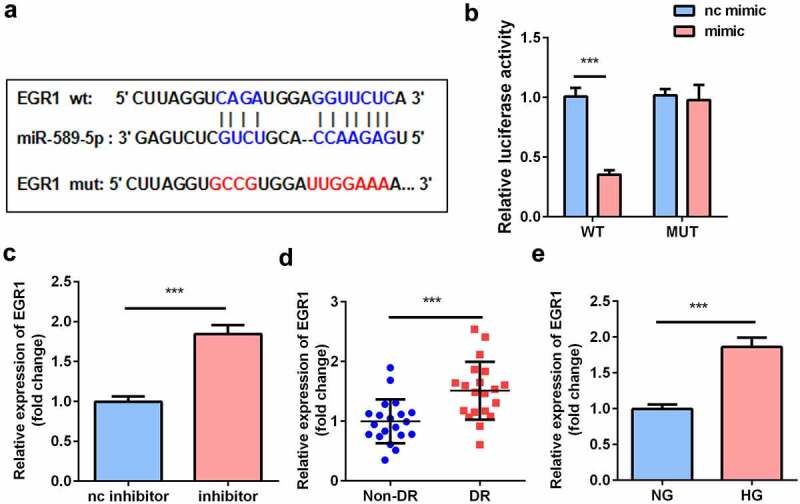
**EGR1 was the target gene of miR-589-5p**. (a) The binding sequences of miR-589-5p and EGR1. (b) Luciferase activities of hRMECs. (c) The transfection efficiency of EGR1. (d) The expressions of EGR1 in the vitreous humor samples from the DR patients (n = 21) and the non-DR people (n = 21). (e) The expressions of EGR1 in hRMECs treated with NG or HG. Each experiment was performed in triplicate. ***P < 0.001. WT, wild type; MUT, mutant type; DR, diabetic retinopathy; EGR1, Early Growth Response Protein 1; hRMECs, human retinal microvascular endothelial cells; HG, high glucose; NG, normal glucose

To further explore how miR-589-5p regulates hRMEC function, the target genes of miR-589-5p were predicted using TargetScan, among which EGR1 was reported to be associated with the progression of DR [14]. Luciferase reporter vectors containing the WT or MUT 3´-UTR of EGR1 were synthesized according to the predicted binding sites with miR-589-5p (Figure 6a). Luciferase activity was significantly lower in hRMECs transfected with EGR1 3´-UTR WT and miR-589-5p mimic (Figure 6b). Moreover, the mRNA expression of EGR1 was significantly reduced in cells transfected with the miR-589-5p inhibitor (Figure 6c). EGR1 levels were both significantly increased in DR patients and HG-treated hRMECs (Figure 6d and e).

### EGR1 aggravated the dysfunctions of hRMECs

**Figure 7.**
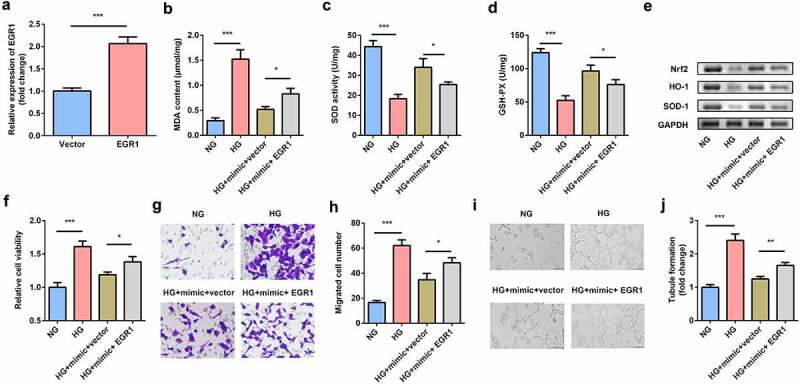
**EGR1 exacerbated oxidative stress and dysfunctions of HG-treated hRMECs**. (a) The expression level of EGR1 after the cells were transfected with EGR1 overexpression plasmid. (b) MDA contents, (c) SOD, and (d) GSH-PX activities in hRMECs were detected using the corresponding kits after the indicated treatments. (e) Western blotting for the proteins of Nrf2, HO-1, and SOD-1. GAPDH was used to normalize the protein levels. (f) MTT assay was performed to determine the cell viability. (g) The migrated cells were fixed and stained after the transwell migration assay. (h) Quantification of G. (i) Images of the tube-like structures were observed under an inverted microscope. (j) Quantification of I. Each experiment was performed in triplicate. *P < 0.05; **P < 0.01; ***P < 0.001. HG, high glucose; MTT, 3-(4,5-dimethylthiazol-2-yl)-2,5-diphenyl tetrazolium bromide; hRMECs, human retinal microvascular endothelial cells; MDA, malondialdehyde; SOD, superoxide dismutase; GSH-PX, glutathione peroxidase

EGR1 was rescued in hRMECs transfected with the miR-589-5p mimic to study the functional correlation between EGR1 and miR-589-5p. Transfection of the EGR1 overexpression plasmid markedly increased EGR1 levels in hRMECs (Figure 7a). As shown in Figure 7b-D, EGR1 significantly facilitated MDA accumulation but decreased SOD and GSH-PX activities. Overexpression of EGR1 reduced the expression of Nrf2, HO-1, and SOD-1 proteins in the cells (Figure 7e). The inhibitory effects of miR-589-5p on viability (Figure 7f), migration (Figure 7g and h), and tube formation (Figure 7i and j) on hRMECs were relieved by EGR1.

## Discussion

DR is the leading cause of visual damage in patients with diabetes [2]. Numerous reports have demonstrated that high glucose levels, hypoxia, inflammation, and neurodegenerative diseases have complicated the treatment of retinal microvascular disease [15]. Therefore, amelioration of retinal microvascular dysfunction is pivotal for DR. circRNAs participate in the progression of DR by sponging miRNAs and further regulating the mRNAs of the target genes [16]. circ-UBAP2 was previously reported to be upregulated in DR [12]. However, the underlying mechanisms remain unclear. Herein, circ-UBAP2 was overexpressed in DR patients and HG-treated hRMECs, which was consistent with the findings of He et al. [12]. Knockdown of circ-UBAP2 ameliorated oxidative stress, viability, migration, and tube formation induced by HG in hRMECs via the miR-589-5p/EGR1 axis. Thence, knockdown of circ-UBAP2 exerted a protective role in DR. To the best of our knowledge, this is the first study to investigate the potential roles of circ-UBAP2 in DR.

circRNAs have been suggested to play vital roles in the pathological process of vascular diseases and regulate proliferation, migration, and apoptosis of the cells [17]. For instance, circRNA microarray analysis showed that hsa_circ_0016070 is correlated with pulmonary arterial hypertension in patients with chronic obstructive pulmonary disease [18]. circDNMT3B overexpression reduces the retinal acellular capillary number and attenuates visual impairment in diabetic rats [19]. In the present study, the expression of circ-UBAP2 was overexpressed in the vitreous humor of DR patients and HG-treated hRMECs, which was consistent with a previous study [20]. Downregulation of circ-UBAP2 suppressed MDA accumulation, promoted the activities of SOD and GSH-PX and the protein levels of Nrf2, HO-1, and SOD-1. These data implied that the knockdown of circ-UBAP2 relieved the oxidative stress induced by HG. In addition, the depletion of circ-UBAP2 inhibited the viability, migration, and tube formation of HG-treated hRMECs and restored cellular functions.

circRNAs serve as competing endogenous RNAs (ceRNAs) to sponge miRNAs [18]. miR-589-5p was predicted and confirmed to be a potential target of circ-UBAP2. To date, miR-589-5p has been mainly investigated in oncogeneses, such as hepatocellular carcinoma [19,20], renal cell carcinoma [21], and laryngocarcinoma [22]. However, it has not been sufficiently explored in DR. Zou et al. [23] confirmed that miR-589-5p targets the oxidative damage gene XPC-rs2229090 C allele in the age-related cataract (ARC), which might increase the risk of ARC. Our data suggest that miR-589-5p mechanically interacts with circ-UBAP2 and is downregulated in DR. In addition, circ-UBAP2 sponged miR-589-5p to exacerbate oxidative stress and enhance cell viability, migration, and tube formation in hRMECs. These findings identified the target miRNA of circ-UBAP2 in DR and provided an in-depth supplement to the previous studies.

EGR1 generally serves as a transcription factor that performs regulatory functions in various diseases [24–26]. An ocular vascular disease-related study demonstrated that EGR1 is expressed in vascular endothelial cells isolated from the active membranes of proliferative diabetic retinopathy (PDR) and proliferative vitreoretinopathy (PVR) patients. This indicates that EGR1 may be involved in inflammatory, angiogenic, and fibrotic responses in proliferative vitreoretinal disorders [27]. Additionally, EGR1 is verified to be upregulated in DR, and hyperglycemia-triggered EGR1 in the retinal endothelium upregulates the expression of downstream pro-thrombotic and pro-inflammatory genes [28]. Likewise, in the present study, EGR1 was overexpressed in vitreous humor samples of DR patients and HG-treated hRMECs, and EGR1 abrogated the inhibitory effects of miR-589-5p on oxidative stress and cell dysfunction induced by HG. These results elucidated the circ-UBAP2/miR-589-5p/EGR1 axis in DR.

## Conclusion

Circ-UBAP2 was upregulated in DR. Loss of circ-UBAP2 relieved HG-induced oxidative stress and dysfunction. Mechanistically, circ-UBA2 sponges miR-589-5p to upregulate the expression of EGR1.The novelty of this article is that we found a potential novel therapeutic strategy for DR progression. While the limitation is that it lacks comments about the clinical significance.

## Data Availability

All the data are available from the corresponding author due to reasonable request

## Research highlights

1. circ-UBAP2 ameliorates oxidative stress and dysfunctions of hRMECs under HG treatment
2. circ-UBAP2spongesmiR-589-5pinhRMECs
3. miR-589-5p targets EGR1 in hRMECs
4. circ-UBAP2/miR-589-5p/EGR1 axis may offer as a potentia therapeutic target for DR
